# Orexin antagonism and substance-P: Effects and interactions on polycystic ovary syndrome in the wistar rats

**DOI:** 10.1186/s13048-023-01168-4

**Published:** 2023-05-05

**Authors:** Somayeh Kouhetsani, Homayoun Khazali, Hassan Rajabi-Maham

**Affiliations:** Department of Animal Sciences and Marine Biology, Faculty of Life Sciences and Biotechnology, Shahid Beheshti University, Tehran, Iran

**Keywords:** Polycystic ovary syndrome, Orexin receptor antagonists, Substance-P receptor antagonist, *Cyp19a1*, Testosterone

## Abstract

**Background:**

Polycystic ovary syndrome (PCOS) is a prevalent endocrine disorder without definitive treatments. Orexin and Substance-P (SP) neuropeptides can affect the ovarian steroidogenesis. Moreover, there are limited studies about the role of these neuropeptides in PCOS. We aimed here to clarify the effects of orexins and SP in PCOS as well as any possible interactions between them.

**Methods:**

For this purpose, the animals (n = five rats per group) received intraperitoneally a single dose of SB-334,867-A (orexin-1 receptor antagonist; OX1Ra), JNJ-10,397,049 (orexin-2 receptor antagonist; OX2Ra), and CP-96,345 (neurokinin-1 receptor antagonist; NK1Ra), alone or in combination with each other after two months of PCOS induction. The blocking of orexin and SP receptors was studied in terms of ovarian histology, hormonal changes, and gene expression of ovarian steroidogenic enzymes.

**Results:**

The antagonists’ treatment did not significantly affect the formation of ovarian cysts. In the PCOS groups, the co-administration of OX1Ra and OX2Ra as well as their simultaneous injections with NK1Ra significantly reversed testosterone levels and *Cyp19a1* gene expression when compared to the PCOS control group. There were no significant interactions between the PCOS groups that received NK1Ra together with one or both OX1R- and OX2R-antagonists.

**Conclusion:**

The blocking of the orexin receptors modulates abnormal ovarian steroidogenesis in the PCOS model of rats. This suggests that the binding of orexin-A and -B to their receptors reduces *Cyp19a1* gene expression while increasing testosterone levels.

## Introduction

Polycystic ovary syndrome (PCOS) is a prevalent endocrinopathy in reproductive-aged females that is characterized by a wide range of endocrine, reproductive, and metabolic disorders. Its significant characteristics include gonadotropin secretion alterations, hyperandrogenism, chronic anovulation, ovarian cysts, and infertility [1, 2]. Furthermore, PCOS, as a polygenic syndrome disorder, has been linked to abnormal expression of genes encoding steroidogenic enzymes [3]. The critical role of steroidogenic enzymes such as steroidogenic acute regulatory protein (*Star*) and aromatase (cytochrome P450, family 19, subfamily A, polypeptide 1; *Cyp19a1*) in PCOS rats has been well demonstrated in previous studies [4, 5]. There are shards of evidence showing that changes in gonadotropin-releasing hormone (GnRH) secretion and gonadotropin ratios affect the gene expression of *STAR* (the steroidogenesis initiator) and *CYP19A1* (an enzyme that converts testosterone to estradiol during steroidogenesis), resulting in an excess of androgen and hyperandrogenemia in PCOS [6]. However, the pathogenesis of PCOS is not completely identified. Therefore, to reduce the complications of PCOS, it is critical to first identify the pathogenesis of the disease and then find the appropriate treatment.

The hypothalamic neuropeptides Orexin-A (OXA) and Orexin-B (OXB) (also recognized as hypocretins) are produced through a proteolytic process from a precursor called pre-pro-orexin. Orexins activate various signaling pathways by binding to their G protein-coupled receptors (GPCRs), which are termed orexin type-1 receptor and orexin type-2 receptor (OX1R and OX2R, or the hypocretin receptors). Both types of receptors are widely distributed inside and outside the central nervous system in a range of species [7, 8]. The binding tendencies of orexin receptors (OXRs) are partially different; OXA binds to OX1R with high affinity, whereas both OXA and OXB bind to OX2R with equal affinity. Orexin receptor-blocking drugs were designed, based on these different receptor affinities. For example, SB-334,867-A and JNJ-10,397,049 are highly selective and potent OX1R- and OX2R-antagonists, respectively [9]. In addition to being involved in food intake, energy metabolism, and sleep-wake rhythm, orexins are involved in reproduction by modulating the hypothalamic-pituitary-ovarian (HPO) relationships [10]. OX1R-expressing GnRH neurons in the hypothalamus are in direct contact with orexin fibers; it appears that the effects of orexins on the HPO axis are mediated by the stimulation of GnRH neurons [11]. In addition, by altering GnRH stimulation, orexins indirectly affect pituitary and ovarian hormone secretions [12]. Previous studies have documented an age-related reduction in the orexin system in rats and mice, either at the expression of the peptide or receptor levels [13, 14]. Iwasa et al. also reported that during development, hypothalamic OX1R gene expression decreased in both male and female rats, while hypothalamic OX2R gene expression increased in males but not in females [15]. In contrast, Yamamoto et al. [16] reported no significant difference in the expression of OX1R and OX2R genes in prepubertal and adult rats. The expression of the OX1R and OX2R genes has also been detected in theca and granulosa cells of rat ovaries [17] and at different stages of the estrous cycle [8]; this emphasizes the high importance of the orexinergic system in ovarian function. The effects of orexins on the regulation of steroidogenic enzymes, the secretion of steroid hormones, and folliculogenesis have been reported. In vitro conditions, orexins can inhibit estradiol secretion by porcine granulosa cells [18]. In addition, orexins decreased progesterone secretions in the luteal cells of rats but did not affect progesterone secretions in the granulosa cells [17]. In contrast, OXA promoted progesterone secretions by regulating the expression of Star, 3β-Hydroxysteroid dehydrogenase, and cytochrome P450 in sheep ovarian granulosa [19]. These studies imply that orexins can affect reproductive functions through the modulation of ovarian steroidogenesis. Furthermore, OXA can play a key regulatory role in granulosa cell development and folliculogenesis in mice by regulating proliferation, which suggests the pro-proliferative function of OXA in these cells [20]. Furthermore, Cataldi et al. [17] found that treatment with OXA increased the expression of OXRs in rat ovaries; these effects were in turn reversed by selective OXR antagonists. Limited data have shown the role of orexins on PCOS; however, other orexigenic factors such as galanin and ghrelin have been described in PCOS patients. Circulating galanin [21] and ghrelin [22] levels are negatively correlated with those of LH (luteinize hormone) and testosterone. A single paper by Yilmaz et al. [23] also reported that OXA levels may be associated with the high serum levels of LH and testosterone in women with PCOS. The exact mechanism by which orexins affect PCOS, however, is not known.

Substance-P (SP) is a bioactive neuropeptide of the tachykinin family that exerts its effects through a GPCR receptor called the neurokinin type-1 receptor (NK1R) or the tachykinin type-1 receptor. NK1R is expressed in different ovarian structures, such as the granulosa cells [24], which suggests an influence of them on ovarian function. As Winiarczyk et al. demonstrated, SP has an inhibitory effect on granulosa cell expansion, cumulus cell expansion, and oocyte growth [25]. SP has a definite modulatory effect on the release of ovarian steroids, but that effect may vary depending on species, age, experimental conditions, and ovarian structure differences. For example, luteal cells, compared to granulosa cells, are more sensitive to the effects of SP [24]. SP can be found in rat ovaries at both the prepubertal [26] and adult stages [27]. The expression of SP and NK1R increases before puberty, suggesting they are initiators of the pubertal stage [28]. CP-96,345, a non-peptide and specific NK1R antagonist (NK1Ra), has demonstrated that SP/NK1R signaling is involved in both physiological and pathological actions, such as the regulation of reproductive function, sex hormone-dependent diseases, and inflammatory diseases [29, 30]. Previous studies reported that SP and Neurokinin-B (NKB), a tachykinin, stimulate GnRH neuronal activity and LH secretions, and inversely, their antagonists can inhibit gonadotropin secretions in several species [31, 32]. Additionally, the dysregulated expression of tachykinins and their receptors in PCOS has been discussed by many reproductive neuroendocrinologists. In this regard, recent clinical studies showed that NKB receptor antagonism ameliorates the symptoms of PCOS patients by modifying LH hypersecretion and hyperandrogenism [33, 34]. However, little is known about how SP affects PCOS. Some researchers hypothesized that the differences in the distribution of SP nerve fibers affected polycystic ovarian function in pigs [35] and women [36], which may play a role in the formation or maintenance of ovarian cysts.

There is evidence supporting the relationship between orexin and SP systems in animal models. Indeed, it has been shown that orexin can activate SP neurons in the ventrolateral periaqueductal gray of mice, where OX1Rs are expressed. Subsequently, SP is released and activates the cascade that causes analgesia [37]. In addition, SP and orexin suppress the KirNB channels in the nucleus basalis neurons of rats, which results in neuronal excitation [38]. SP has similar behavioral effects as orexin in the anterior paraventricular nucleus of the thalamus (aPVT), likewise, a local NK1Ra blocks orexin-induced ethanol drinking in the aPVT of rats [39]. Moreover, orexin and SP suppress food and water intake when administrated in the ventricles of rats, suggesting that these peptides may have similar effects in several brain areas [39]. The idea that orexin and SP may interact in PCOS is also suggested by findings showing the presence of their receptors throughout the HPO axis and particularly in the ovary [10, 40]. It has also been established that orexin and SP have an effect on HPO relationships and ovarian and testicular steroidogenesis in animals like rats and mice. Therefore, given the effects of other orexigenic factors and tachykinin on PCOS, we designed a study using OXR and NK1R antagonists to examine how endogenous orexin and SP, or probably their interaction, affect PCOS conditions.

Considering the above literature, we hypothesized that OXR and NK1R antagonists would likely have modulating effects in the PCOS model of rats. As such, the organ coefficient, ovarian histology, serum concentrations of dehydroepiandrosterone sulfate (DHEAs) and testosterone, and the expression of the *Star* and *Cyp19a1* genes in the ovarian tissue were examined.

## Materials and methods

### Materials

Estradiol Valerate (EV) (Aburaihan Pharmaceutical, Iran), SB-334,867-A (orexin-1 receptor antagonist: OX1Ra, Tocris Bioscience, USA), JNJ-10,397,049 (orexin-2 receptor antagonist: OX2Ra, Tocris Bioscience, USA), and CP-96,345 (NK1Ra, Tocris Bioscience, USA) were used in the present study. The respective antagonists were dissolved in a mixture of saline and Dimethyl Sulfoxide. The solutions were immediately prepared before intraperitoneal (IP) injection.

### Animals and experimental design

Forty-five pubertal female Wistar rats (45–48 days old and 180–200 g) were randomly allocated to different cages and maintained under laboratory standard conditions, including a temperature- and humidity-controlled environment (22 °C and 50%, respectively), a light/dark period of 12 h, and free access to a normal diet and water. Experimental procedures were carried out under international standards for the care and use of laboratory animals. In addition, the ethics committee of Shahid Beheshti University confirmed this experimental protocol with the code IR.SBU.REC.1400.074. The rats were acclimated to the laboratory environment for one week before the experiments. All the animals were examined daily by vaginal cytology for regular estrous cycles. The estrous cycle was evaluated by the relative ratio of cells, including leukocytes, epithelial, and cornfield cells, in the vaginal smear using the optical microscope. For this experiment, the rats with at least two consecutive 4-day estrous cycles were chosen. The randomly assigned rats to nine groups are listed in Table 1 (n = 5 rats per group):

**Table 1:** Experimental groups and drug administration

Groups	Received solutions
1	Control (only received vehicle)
2	PCOS (EV, 4.0 mg/kg); IM
3	PCOS + OX1Ra (1.0 mg/kg); IP
4	PCOS + OX2Ra (1.0 mg/kg); IP
5	PCOS + OX1Ra (1.0 mg/kg) + OX2Ra (1.0 mg/kg); IP
6	PCOS + NK1Ra (1.0 mg/kg); IP
7	PCOS + OX1Ra (1.0 mg/kg) + NK1Ra (1.0 mg/kg); IP
8	PCOS + OX2Ra (1.0 mg/kg) + NK1Ra (1.0 mg/kg); IP
9	PCOS + OX1Ra (1.0 mg/kg) + OX2Ra (1.0 mg/kg) + NK1Ra (1.0 mg/kg); IP

PCOS: polycystic ovary syndrome, IM: intramuscular injection, IP: intraperitoneal injection, OX1Ra: orexin-1 receptor antagonist, OX2Ra: orexin-2 receptor antagonist, NK1Ra: neurokinin-1 receptor antagonist.

### Induction of the PCOS model and drug administration

**Fig. 1:**
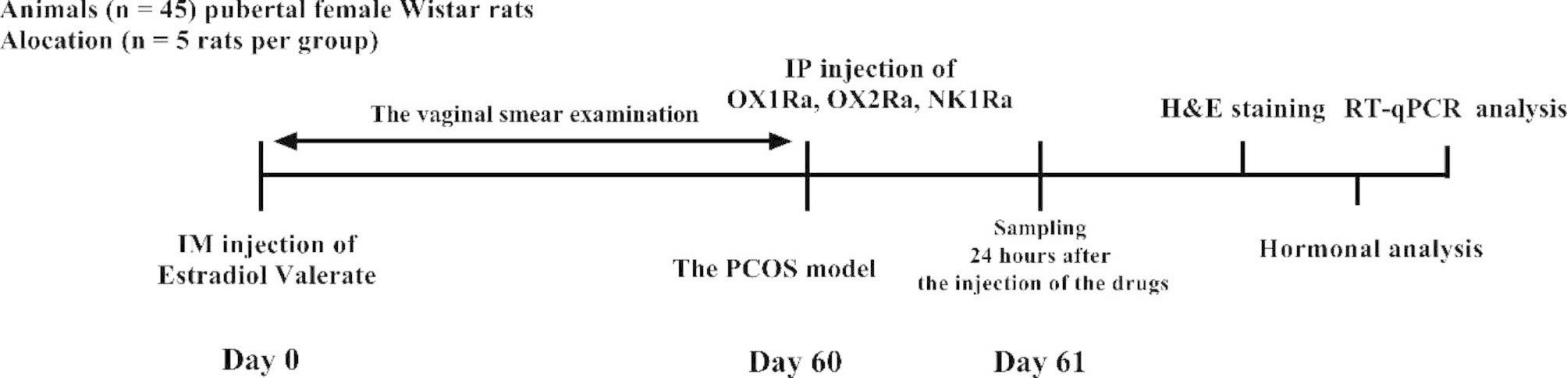
This figure shows the experimental procedures of present study. SB-334,867-A (OX1Ra), JNJ-10,397,049 (OX2Ra), and CP-96,345 (NK1Ra), alone or in combination with each other, were intraperitoneally injected after two months of PCOS induction. The samples were collected 24 h after the injection of the drugs, for future investigations. IM: intramuscular injection, PCOS: polycystic ovary syndrome, IP: intraperitoneal injection, OX1Ra: orexin-1 receptor antagonist, OX2Ra; orexin-2 receptor antagonist, NK1Ra: neurokinin-1 receptor antagonist, H&E staining: hematoxylin and eosin staining, RT-qPCR analysis: Quantitative real-time PCR analysis

EV diluted with sesame oil (0.2 ml, as a vehicle) was intramuscularly administered at a single dose of 4.0 mg/kg on an estrous day [1]. During the PCOS induction, vaginal swabs were collected to confirm the period of persistent vaginal cornification. Two months after treatment, the animals characterized by disorders of the estrous cycle and prolonged diestrous, which was considered for the PCOS model. After this period, the rats received IP injections of SB-334,867-A (OX1Ra, 1 mg/kg, 0.1 ml), JNJ-10,397,049 143 (OX2Ra, 1 mg/kg, 0.1 ml), and CP-96,345 (NK1Ra, 1 mg/kg, 0.1 ml) in a single dose manner according to group order. Antagonist injections were performed at 20-minute intervals, in groups that received more than one antagonist. Based on previous rodent studies [8, 41, 42], the dosages of OXR and NK1R antagonists were chosen to block these receptors. The protocol of this experiment is shown in Fig. 1.

### Sampling

The samples were collected 24 h after the injection of the drugs. The rats were anesthetized (IP injection of 50 mg/kg Ketamine and 7 mg/kg Xylazine) at the diestrus stage, and the blood samples were collected from the heart, and centrifuged (at 3500 g for 15 min). The serum samples were separated and kept at -20 ◦C for future use. The animals were euthanized by carbon dioxide inhalation and their ovaries were immediately removed and cleaned with ice-cold saline. The left ovary was fixed in 10% formalin for histological studies, and the right ovary was immersed in liquid nitrogen and stored at -80 ◦C for further analysis of gene expression [1, 43].

### Body and ovarian weight determination

The initial and final body weights of the animals were monitored in all of the experimental groups. Moreover, the ovarian weight of the rats was measured immediately following sacrificing, and the organ coefficient was calculated by dividing the ovarian weight of the animals by their body weight [44].

### Histopathological examination

Paraffin-embedded ovarian tissues were sliced into 5-µm-thick sections through a microtome. The ovarian sections were dehydrated in 70, 80, 90, and 100% ethanol, deparaffinized in xylene, hydrated in 100, 90, 80, and 70% ethanol, and stained with hematoxylin and eosin (H&E) (Sigma-Aldrich, USA). After clearing with fresh xylene, histological changes were observed under a Nikon model YS100 microscope (Nikon, China). The different types of follicles, especially cystic follicles and corpus luteum, were identified. In addition, the number of follicular cysts was counted per ovary Sects. [43, 45].

### Hormonal analysis

The concentrations of serum DHEAs and testosterone were measured by the DHEAs [^125^I] radioimmunoassay (RIA) Kit (Izotop Co., Hungary) and the Testosterone [^125^I] RIA Kit (Izotop Co., Hungary). The sensitivity, intra-, and inter-assay coefficients of variation were 0.064 µmol/l, 3.05%, and 5.32% for DHEAs, respectively, and they were 0.21 nmol/l, 7.3%, and 12%, respectively, for testosterone. The standard curves were used to estimate the DHEAs and testosterone concentrations.

### Total RNA extraction and cDNA synthesis

Total RNA was cautiously extracted from the right ovary of rats in the experimental groups using the Total RNA Extraction Kit (Parstous Co., Iran). The extracted RNA was then treated with DNase I (Parstous Co., Iran). The concentration of extracted RNA was determined using a Synergy HTX Multi-Mode Reader (BioTek Co., USA) with a ratio of absorbance at 260/280 nm. The RevertAid single-strand cDNA Synthesis Kit (Parstous Co., Iran) was used to synthesize cDNA from 1 µg of extracted RNA. The steps mentioned above were conducted following the instructions of the Parstous Kit.

### Quantitative real-time PCR

The sequences of the forward and reverse primers for target genes were documented in Table 2. Oligo primer analysis software was used to design the primers. Quantitative real-time PCR (RT-qPCR) was used to evaluate the relative expression of genes with RealQ Pus 2x Master Mix Green (AMPLIQON, Denmark) using the Step One Plus Real-Time PCR System (Applied Biosystems, USA). For each given transcript, RT-qPCR was performed in a duplicate manner with a total volume of 25 µl according to the following three-step program: one cycle of 95 °C for 15 min; and a whole number of 40 cycles at 95 °C for 20 s, 60 °C for 30 s, and 72 °C for 30 s. Cycle threshold was obtained for all genes and normalized to glyceraldehyde-triphosphate-dehydrogenase (*Gapdh*) as a reference gene. The 2^−ΔΔCT^ method was calculated to determine the relative expression of each target gene [46].

**Table 2:** The following is a summary of information on the primer sequences of related genes:

Gene name	Gene Bank accession number	Primer sequence (5’ -3’)	Amplicon size (bp)
*Star*	NM_031558.3	F-TGTACCAAGCGTAGAGGTTC R-GCATCTCCCCAAAGTGTG	64
*Cyp19a1*	NM_017085.3	F-GAAAACTTCATTAACGAGAGCC R-ATGACCAAGTCCACGACAG	52
*Gapdh*	NM_017008.4	F-AACGACCCCTTCATTGACCT R-GGTTTCCCGTTGATGACCAG	119

The relative expressions of genes were normalized to the housekeeping gene. F: forward, R: reverse, *Star*: steroidogenic acute regulatory protein, *Cyp19a1*: cytochrome P450, family 19, subfamily a, polypeptide 1, *Gapdh*: glyceraldehydes-3-phosphate dehydrogenase.

### Statistical analysis

The statistical analysis of data was done by IBM SPSS 22 software. The normality of variables was examined by the Shapiro-Wilk test. A one-way ANOVA with the post-hoc Tukey HSD was used to determine the differences between treatment groups. The differences at *P* ≤ 0.05 were considered statistically significant. All graphs were drawn using GraphPad Prism 9 software. The results were presented as the median (min, max).

## Results

### The effects of OXR and NK1R antagonism on body and ovarian weight

**Fig. 2:**
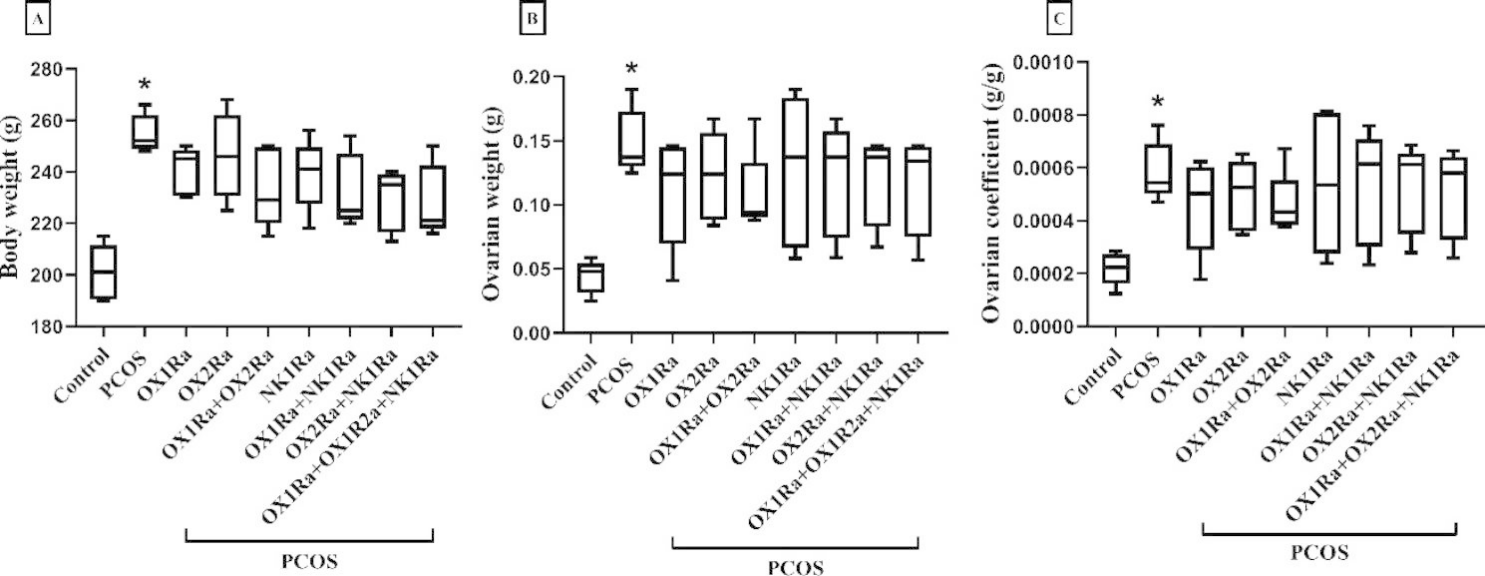
The effects of OXR and NK1R blockade on (A) body weight, (B) ovarian weight, and (C) ovarian coefficient in the EV-induced PCOS rats. PCOS: polycystic ovary syndrome, OX1Ra: orexin-1 receptor antagonist, OX2Ra: orexin-2 receptor antagonist, NK1Ra: neurokinin-1 receptor antagonist. The data are presented as median (min, max). *P ≤ 0.05 compared to the control group by the Tukey’s test

The results showed that a single dose of EV significantly increased body weight, ovarian weight, and ovarian coefficient in the PCOS control group compared to the control group (*P* = 0.001, *P* = 0.003, and *P* = 0.037, respectively). However, neither OXR nor NK1R antagonists had a substantial effect on these parameters in any of the PCOS groups (Fig. 2A–C).

### The effects of OXR and NK1R antagonism on ovarian morphology

**Fig. 3:**
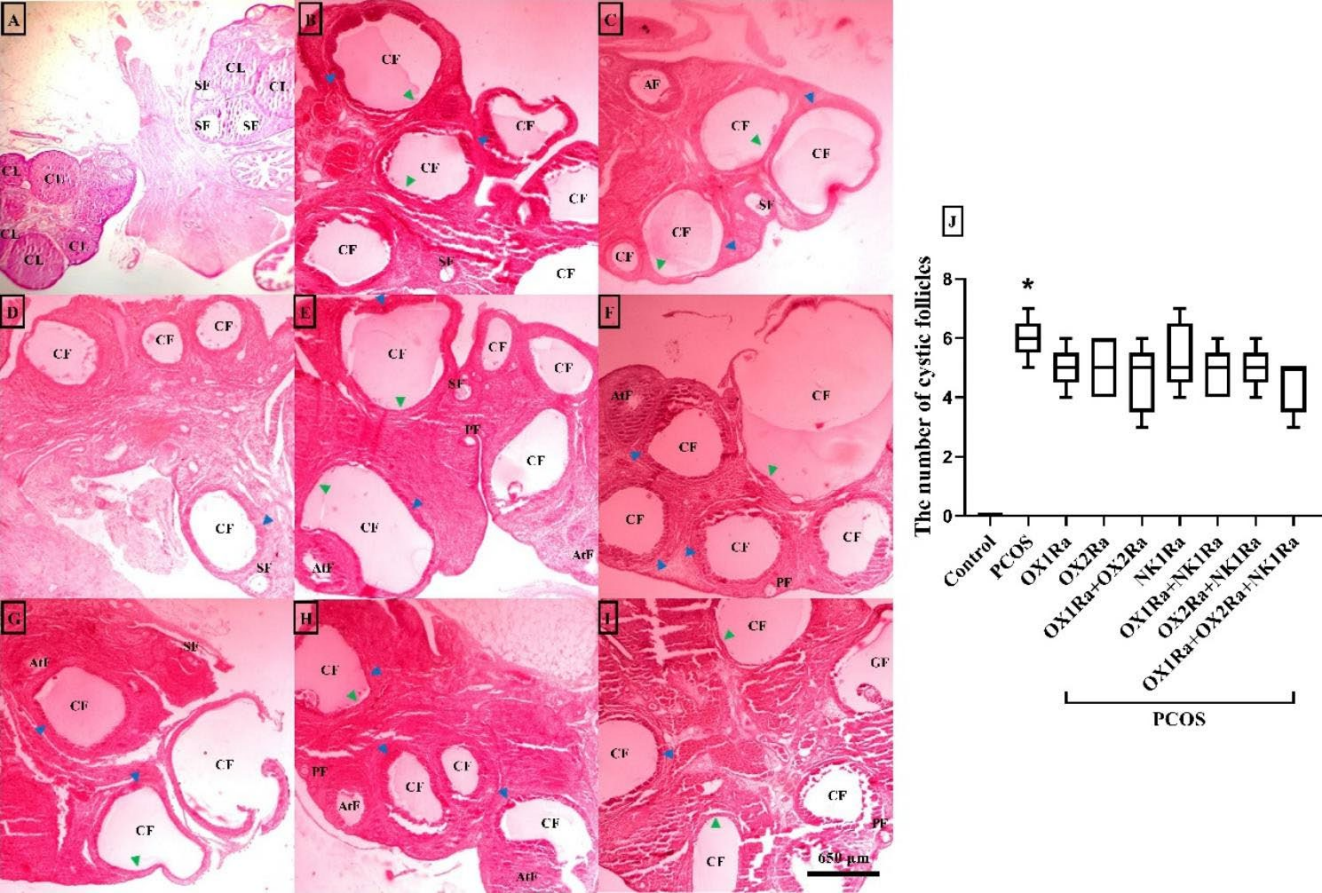
Histological analysis of the ovarian rats (H&E staining). (A) Control group, (B) PCOS control group, (C) PCOS + OX1Ra, (D) PCOS + OX2Ra, (E) PCOS + OX1Ra + OX2Ra, (F) PCOS + NK1Ra, (G) PCOS + NK1Ra + OX1Ra, (H) PCOS + NK1Ra + OX2Ra, (I) PCOS + OX1Ra + OX2Ra + NK1Ra, and (J) The number of cystic follicle. CL: corpus luteum, PF: primary follicle, SF: secondary follicle, AT: antral follicle, GF: graafian follicle, AtF: atretic follicle, CF: cystic follicle, PCOS: polycystic ovary syndrome, OX1Ra: orexin-1 receptor antagonist, OX2Ra: orexin-2 receptor antagonist, NK1Ra: neurokinin-1 receptor antagonist, Green triangle: granulosa cell layer, Blue triangle: theca cell layer. Magnified ×40. The scale bars represent 650 μm. The data is presented as the median (min, max). *P ≤ 0.05 compared to the control group by the Tukey’s test

The PCOS model was confirmed after observing the irregular cycle, which was mostly in the estrous, and following that, histological investigations were performed. In this regard, H&E staining demonstrated normal ovarian morphology with several corpus luteum and developing follicles in the control group (Fig. 3A). In the PCOS control group, there were significantly more enlarged cystic follicles with layers of hyperplasia theca cells and layers of degenerating granulosa cells than in the vehicle group (*P* = 0.001) (Fig. 3J). IP injection of antagonists, either alone or in combination with each other, hardly reversed the number of ovarian cysts in the PCOS groups (Fig. 3B-J). Notably, the majority of the PCOS groups did not show corpus luteum formation.

### The effects of OXR and NK1R antagonism on the hormone serum levels

**Fig. 4:**
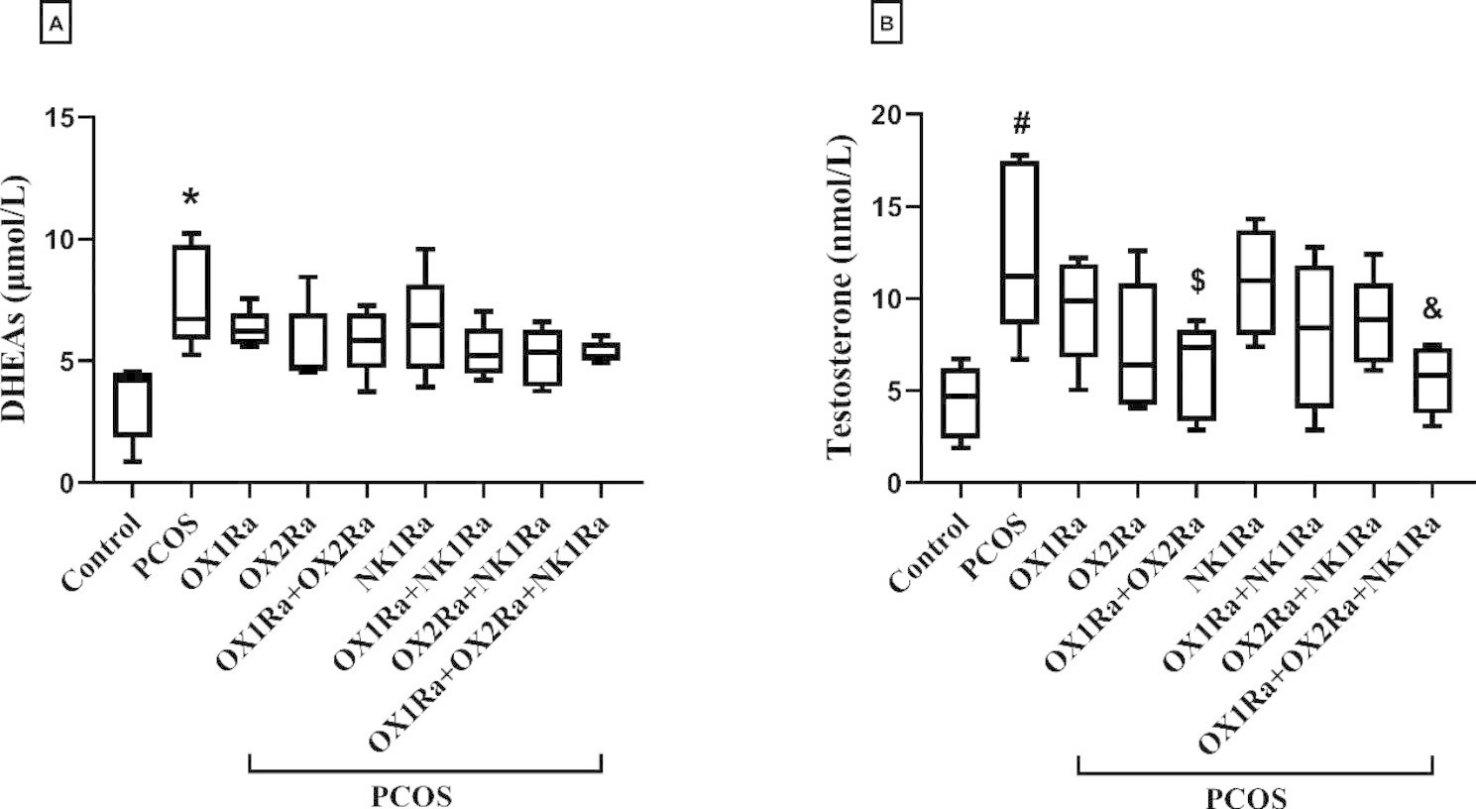
The effects of OXR and NK1R blockade on the hormone serum levels in the EV-induced PCOS rats. (A) DHEAs and (B) Testosterone. DHEAs: dehydroepiandrosterone sulfate, PCOS: polycystic ovary syndrome, OX1Ra: orexin-1 receptor antagonist, OX2Ra: orexin-2 receptor antagonist, NK1Ra: neurokinin-1 receptor antagonist. The data are presented as median (min, max). *P ≤ 0.05 and ^#^*P* ≤ 0.05 compared to the control group by the Tukey’s test. ^$^*P* ≤ 0.05 and ^&^*P* ≤ 0.05 compared to the PCOS control group by the Tukey’s test

In comparison to the control group, both DHEAs and testosterone levels were substantially increased in the PCOS control group (*P* = 0.002 and *P* = 0.005, respectively) (Fig. 4A, B). The testosterone levels, but not DHEAs, were significantly reduced with the co-administration of OX1Ra + OX2Ra as well as concurrently with NK1Ra in the PCOS groups compared to the PCOS control group (*P* = 0.050 and *P* = 0.026, respectively) (Fig. 4A, B). In other PCOS groups, the administration of OX1Ra, OX2Ra, and NK1Ra either separately or simultaneously did not exert meaningful differences in both DHEAs and testosterone serum levels compared to the PCOS control group. Moreover, in OX1Ra-, OX2Ra-, and OX1Ra + OX2Ra-treated PCOS groups, the IP injection of NK1Ra had a negligible interaction with both hormones compared to their counterparts (Fig. 4A, B).

### The effects of OXR and NK1R antagonism on the relative gene expression of the ***Star*** and ***Cyp19a1*** genes

**Fig. 5:**
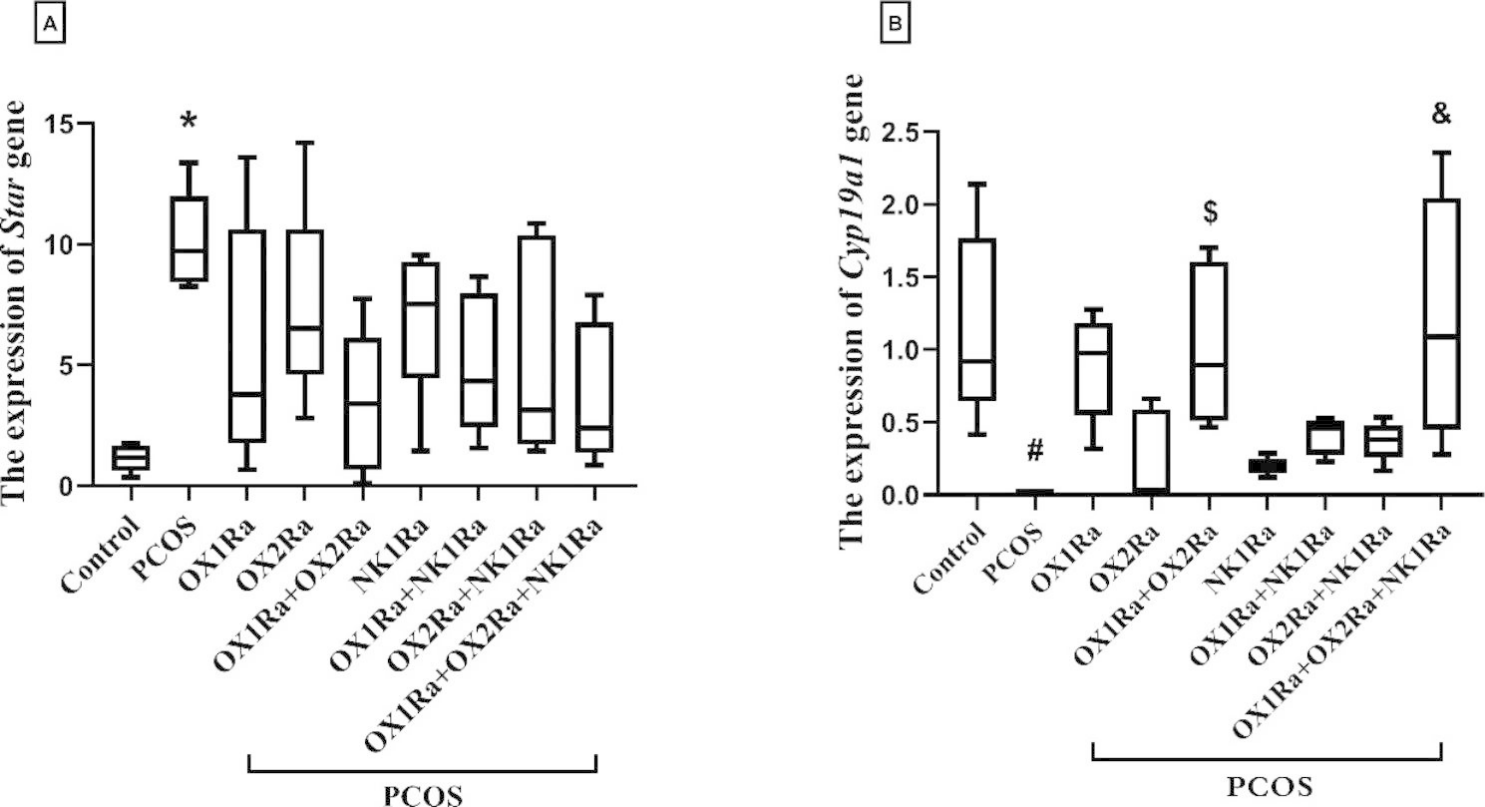
The effects of OXR and NK1R blockade on the relative expression of the steroidogenic genes in the EV-induced PCOS rats. (A) The expression of the *Star* gene and (B) The expression of the *Cyp19a1* gene. The expression of target genes was normalized by the *Gapdh* gene in each sample. *Star*: steroidogenic acute regulatory protein, *Cyp19a1*: cytochrome P450 Family 19 Subfamily A Member 1, PCOS: polycystic ovary syndrome, OX1Ra: orexin-1 receptor antagonist, OX2Ra: orexin-2 receptor antagonist, NK1Ra: neurokinin-1 receptor antagonist. The data are presented as median (min, max). *P ≤ 0.05 and ^#^*P* ≤ 0.05 compared to the control group by the Tukey’s test. ^$^*P* ≤ 0.05 and ^&^*P* ≤ 0.05 compared to the PCOS control group by the Tukey’s test

As seen in Fig. 5, there was a significant increment in the expression of *Star* (*P* = 0.005), whereas there was a significant decrement in the expression of *Cyp19a1* between the PCOS control and control groups (*P* = 0.005) (Fig. 5A, B).

The co-treatment of OX1Ra + OX2Ra or together with NK1Ra in the PCOS groups resulted in a marked elevation in the expression of *Cyp19a1* compared to the PCOS control group (*P* = 0.018 and *P* = 0.003, respectively) (Fig. 5B), while the expression of *Star* seems to be unaffected (Fig. 5A). In other PCOS groups that received separate or concurrent injections of OX1Ra, OX2Ra, and NK1Ra, increased *Star* expression and decreased *Cyp19a1* expression were observed when compared to the PCOS control group, but these changes were negligible. Furthermore, when NK1Ra was simultaneously injected with one or both OX1R- and OX2R-antagonists, neither *Star* nor *Cyp19a1* expression showed any evidence of a significant interaction (Fig. 5A, B).

### Pearson’s correlation coefficient between testosterone levels and the expression of the ***Star*** or ***Cyp19a1*** genes

**Fig. 6:**
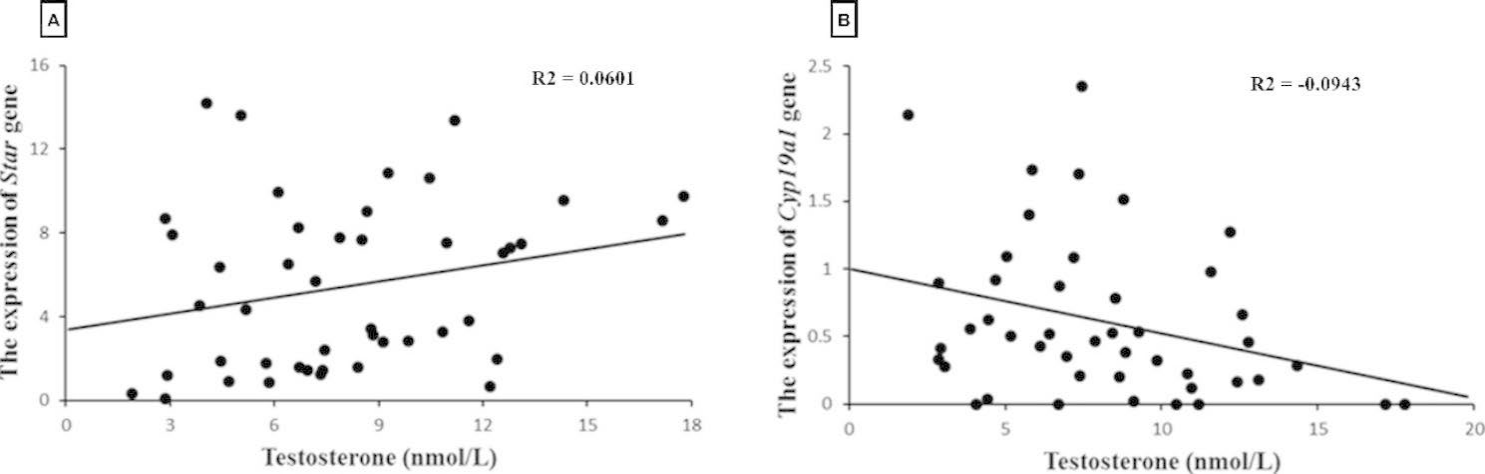
Pearson’s correlation coefficient between the expression of *Star* and testosterone as well as *Cyp19a1* and testosterone. The expression of *Star* was not correlated with testosterone levels. The expression of *Cyp19a1* was negatively correlated with testosterone levels. *Star*: steroidogenic acute regulatory protein, *Cyp19a1*: cytochrome P450 Family 19 Subfamily A Member 1. These relationships are presented as R^2^ = 0.0601 and R^2^ = -0.0943 according to sections A and B

Pearson’s correlation reported an insignificant positive relationship between the expression of *Star* and testosterone levels in different groups as well as a significant relationship between the expression of *Cyp19a1* and testosterone, meaning that there was a negative correlation between decreased *Cyp19a1* expression compared to increased testosterone levels (*P* = 0.040) (Fig. 6).

## Discussion

Here, for the first time, we investigated how PCOS symptoms in Wistar rats might be influenced by blocking the binding of orexins and SP to their receptors using OXR and NK1R antagonists.

The PCOS model was successfully induced two months after EV administration, as confirmed by weight examination, H&E staining, RIA, and qRT-PCR findings. As such, we clearly showed an increase in body and ovarian weight, an increase in the ovarian coefficient, and several bulky follicular cysts with altered theca and granulosa layers. The ovarian coefficient increment in the PCOS group could be related to a higher elevation in ovarian weight compared to body weight, as demonstrated by other authors [44, 47], which is probably due to an increase in the ovarian volume and the number of cysts [48]. Follicular cysts were not significantly affected by OXR antagonists. Since a great reduction in the number of these cysts was observed when OXR antagonists were administered concurrently; it seems that orexins are involved in polycystic ovary morphology. On the other hand, increased testosterone levels may play a role in cyst formation, which is decreased following the injection of orexin receptor antagonists. As Morales-Ledesma et al. [49] and Anesetti and Chávez-Genaro [50] reported, testosterone results in the formation of cystic structures. These studies provide evidence that supports the idea that ovarian cyst formation depends on changes in steroid hormones. There is also evidence to suggest that orexin-A stimulates GnRH, LH, and testosterone secretions in male and female rats [51, 52]. Therefore, it can be assumed that orexin contributed to the formation of ovarian cysts by increasing testosterone levels.

It is well documented that dysregulated ovarian steroidogenesis is involved in the initiation and development of PCOS in both animals and women [4, 5]. We have also shown that the polycystic ovary of the rats is consistent with hyperandrogenism-related steroidogenic defects, including an increase in DHEAs and testosterone levels and the expression of *Star* as well as a decrease in the expression of aromatase. Furthermore, this is the first report to show that OX1Ra + OX2Ra treatment reversed the expression of *Cyp19a1* and testosterone levels in the PCOS model of rats, implying that orexins differentially affect steroidogenesis and contribute to the pathogenesis of PCOS. It should be noted that decreased levels of testosterone were higher than those of DHEAs, and the expression of *Cyp19a1* had more changes than the *Star* gene. This shows how orexigenic peptides contribute to the hyperandrogenism associated with PCOS, which, based on these results, we presume is mediated by *Cyp19a1* and testosterone factors.

It is not yet clear how these positive effects are mediated in the PCOS model, although studies are showing that orexins and OXR antagonists have an effect on steroidogenesis in various models. In females, the varied effects of these compounds on uterine steroidogenesis in cyclic and pregnant pigs have been documented [53–56]. Kiezun et al. [53] and Rytelewska et al. [56] reported that OXA and the antagonist of its receptor, SB-334,867-A, have different effects on the expression of the *Star*, *Cyp11a1*, *Hsd3b1*, *Cyp17a1*, and *Cyp19a3* genes, as well as on progesterone, testosterone, and estradiol hormone secretions. The above-mentioned factors were also determined by other authors in the presence of OXB and the antagonist of its receptor, JNJ-10,397,049 [54, 55]. However, Grasselli et al. [57] rejected the impact of OXB on this process in swine ovarian follicles. Collectively, they claimed that the observed effects could depend on the type of tissues, the dose of drugs, the days of pregnancy, or the estrous cycle [53–57]. These studies presented the finding that the applied OXR antagonists have not completely blocked the effects of orexins on factors related to steroidogenesis. This could be caused by the fact that the receptors were not fully blocked because there is competition between the ligand and antagonist for concurrent binding to the receptor. However, when OXR antagonists were individually added to the cultures, they were able to affect gene expressions and steroid secretions, which probably acted through the unblocked receptor [53–56]. On the contrary, our assessed factors did not significantly alter when OX1Ra or OX2Ra were separately injected; however, the combination of both selective OXR antagonists resulted in additive antagonistic effects. In males, Liguori et al. [58] and Assisi et al. [59] revealed that aromatase activity may be indirectly inhibited by locally secreted OXA interacting with OX1R and may prevent in vitro conversion of testosterone to estrogen in the testes of alpacas and dogs, respectively. They also stated that the OX1R blocker, SB-408,124, significantly reduces this effect [58, 59]. Joshi and Singh [60] discovered that OXA directly affects the regulation of testicular steroidogenesis in adult mice using an OX1R antagonist similar to ours in both in vivo and ex vivo experiments. Different from our data, this report showed degenerative changes in steroidogenesis following the antagonist treatment. According to the above evidence, orexins and OXR antagonists could affect testosterone secretion, which might be explained by the regulation of upstream pathway genes such as *Star*, *Cyp11a1*, and *Cyp17a1*, or the inhibition of the aromatase gene. These studies are partially in accordance with our findings. We described a stimulatory effect of OXR antagonists on the expression of *Cyp19a1*, which probably reduced testosterone levels. The insignificant change in *Star* expression, on the other hand, failed to explain how testosterone levels decreased. This shows that other genes may be involved in this process, but this work was limited by the lack of examination of them. However, the differences in experimental models, especially between species, target organs, and hormonal states, should be considered when the effects of orexins are examined.

The activation of orexin receptors in GnRH neurons stimulates gonadotropin secretions and subsequently, steroid hormone secretions in rodents [7, 12]. Furthermore, Silveyra et al. [8] confirmed a decrease in the gonadotropin-proestrous release following OX1Ra or OX2Ra administration, alone or in combination with each other, in rats. They suggested that the effects of OXR antagonists, which are the same as our antagonists, were mostly limited to the two upper branches of the HPG axis [8]. Therefore, we hypothesized that OXR antagonists decreased androgen secretions in theca cells by reducing the GnRH pulse frequency and pulsatile LH release. As a result, the stimulatory effect of androgen on FSH and subsequently, on the expression of *Cyp19a1* was reduced in the polycystic ovary of rats. It should be determined whether these effects were caused by an intra-ovarian change or a blockade of OXRs in ovarian cells. Whether they were mediated by the blockade of OXRs at other levels of the HPO axis, either by inhibiting the secretion of hypothalamic orexins or pituitary gonadotropins.

The findings suggest that orexigenic factors such as ghrelin and galanin are involved in PCOS. Excess androgens in PCOS patients may directly result in ghrelin reduction [61]. Likewise, decreased ghrelin affects the hyperstimulation of gonadotropic and ovarian cells in this syndrome [62]. It was also suggested that the ovarian expression of ghrelin might be changed in women with PCOS [62]. However, Daghestani et al. reported no difference in ghrelin levels in PCOS patients compared with controls [63]. Recently, Azin et al. [1] showed that IP injection of galanin in PCOS rats induced decreased LH and testosterone levels and increased FSH levels. They also found that the expression of the *Star* and *Cyp19a1* genes was significantly reversed with galanin treatment [1]. Altinkaya reported a negative correlation between galanin and LH levels in PCOS women. Likewise, LH levels could not be inhibited due to reduced galanin [21]. Besides these, Iwasa et al. [64] demonstrated that the expression of the pre-pro-orexin gene remained unchanged in 5α-dihydrotestosterone-induced PCOS rats. On the one hand, it is unclear how orexin levels might change in the PCOS model of rats, but on the other hand, Yilmaz et al. [23] reported that serum OXA levels decreased in PCOS-affected women. According to their hypothesis, the low levels of OXA in PCOS women may be an early stage of OXA signaling that protects the patients from the unfavorable effects of OXA in PCOS [23]. Thus, the serum levels of orexin in different PCOS models are needed to better understand the effects of orexin on PCOS.

This is the first description of the effect of NK1Ra and its interaction with OXR antagonists on the PCOS model of rats. Interference in the binding of SP to its receptor failed to overcome the abnormalities observed in the EV-induced PCOS rats because the PCOS symptoms were insignificantly changed after treatment with the NK1Ra, CP-96,345.

A clinical study in women with PCOS revealed that fezolinant treatment, an NKB receptor antagonist, was able to normalize the LH-to-FSH ratio and decrease hyperandrogenism. However, there were no differences in either menstrual cycle regulation or follicle counts [33]. Another study reported that the expression of the *TAC1* and *TACR1* genes was increased in the ovarian cells of women with PCOS [65]. Indeed, dysregulated expression of tachykinins and their receptors is probably associated with fertility problems in these patients [65], which is contrary to our findings. In agreement with this study, the effect of the NKB receptor antagonist, MLE4901, on reproductive defects in the PCOS model of mice was refuted by Sucquart et al. [66], which probably supports the idea that tachykinin signaling differs between species. However, these authors hypothesized that the development of the androgen receptor-mediated reproductive PCOS-like traits is not mediated via the NK3R in the Dihydrotestosterone (DHT)-induced PCOS mouse model, or NK3R may indeed be involved, but the antagonist was unable to overcome the chronically elevated DHT of the model [66]. Thus, the differences in the experimental conditions should not be ignored. Furthermore, animal studies have shown that NKB antagonism provides new capabilities for the management of endocrine-based syndromes such as PCOS by manipulating high GnRH/LH pulsatility [67]. Fergani et al. [32] rejected any critical role for SP in the regulation of reproductive function, as they found no NK1R in GnRH- or Kisspeptin-containing neurons in the ovine hypothalamus. However, other laboratory findings reported that SP can stimulate GnRH neurons and gonadotropin secretion in animals, including rats and mice [68, 69]. Hence, NK1R antagonists may affect reproductive hormone secretion [31]. A possible explanation for our findings could be related to several items. First, NK1R is not involved in this model. Then, its selective antagonist was not able to overcome this chronic model, or its dose was not sufficient. Therefore, we suggest that a longer period of treatment may be required, since a short period of this antagonist was not effective enough to reveal significant effects.

The results of the present study show that there was no interaction between SP and orexin signaling pathways in the PCOS model of rats. However, we observed that NK1Ra in combination with one or both OX1R- and OX2R-antagonists resulted in a higher increase and decrease in measured parameters. Since our study was limited to the use of antagonists for the relevant receptors, the interaction between SP and orexins cannot be evaluated with only these measured parameters. Therefore, modeling these neuropeptides and their inhibitors is necessary for future research. As mentioned before, we assumed that the binding of orexins to their receptors in the PCOS model of rats prevented successful steroidogenesis. However, more research is required to confirm this. Accordingly, a longer course of treatment with different doses of OXR antagonists is suggested. Another limitation of the study was not being able to investigate the gene and protein expression of OX1R, OX2R, and NK1R. In the future, we will explore the expression of the OXR and NK1R genes and proteins in PCOS conditions at different levels of the HPO axis.

## Conclusion

Compared with OX1R- and OX2R-antagonists, NK1Ra appears to have less effect on PCOS symptoms, which suggests a strong function of orexins in this disease. The effects of OXR antagonists on testosterone levels and changes in the *Cyp19a1* (aromatase) gene expression suggest that the binding of orexins to their receptors plays a role in the development of PCOS. Therefore, OXR antagonists may affect the treatment of PCOS and/or the course of the disease. Further studies are required to verify the effects of OXR and NK1R antagonists on PCOS as well as to clarify their mechanisms.

## Data Availability

The datasets used and/or analyzed in the current study are available from the corresponding author upon reasonable request.

## Abbreviations

PCOS: Polycystic ovary syndrome
*Star*: steroidogenic acute regulatory protein
*Cyp19a1*: cytochrome P450, family 19, subfamily A, polypeptide 1
GnRH: Gonadotropin-releasing hormone
OXA: Orexin-A
OXB: Orexin-B
OX1R: orexin type-1 receptor
OX2R: orexin type-2 receptor
OXRs: orexin receptors
HPO: Hypothalamic-pituitary-ovarian
LH: Luteinizing hormone
SP: Substance-P
NK1R: Neurokinin type-1 receptor
NK1Ra: NK1R antagonist
NKB: Neurokinin-B
aPVT: Anterior paraventricular nucleus of the thalamus
DHEAs: dehydroepiandrosterone sulfate
EV: Estradiol Valerate
OX1Ra: Orexin-1 receptor antagonist
OX2Ra: Orexin-2 receptor antagonist
IP: Intraperitoneal
IM: intramuscular
H&E: Hematoxylin and eosin
RIA: Radioimmunoassay
RT-qPCR: Quantitative real-time PCR
*Gapdh*: Glyceraldehyde-triphosphate-dehydrogenase
DHT: Dihydrotestosterone
